# Muscle-Based Pharmacokinetic Modeling of Marrow Perfusion for Osteoporotic Bone in Females 

**DOI:** 10.1155/2014/620925

**Published:** 2014-06-09

**Authors:** Heather Ting Ma, James F. Griffth, Ping-Chung Leung

**Affiliations:** ^1^Department of Electronic and Information Engineering, Harbin Institute of Technology Shenzhen Graduate School, Room 205C, C Building, HIT Campus, Shenzhen University Town, Xili, Nanshan District, Shenzhen 518055, China; ^2^Department of Imaging and Interventional Radiology, Prince of Wales Hospital, The Chinese University of Hong Kong, Shatin, Hong Kong; ^3^Jockey Club Centre for Osteoporosis Care and Control, Public Health School, Prince of Wales Hospital, The Chinese University of Hong Kong, Shatin, Hong Kong

## Abstract

The pharmacokinetic model has been widely used in tissue perfusion analysis, such as bone marrow perfusion. In the modeling process, the arterial input function is important to guarantee the reliability of the fitting result. However, the arterial input function is variable and hard to control, which makes it difficult to compare results across different studies. The purpose of this study was to establish a muscle-based pharmacokinetic model for bone marrow perfusion without using arterial input function. Erector spinae muscle around the vertebral body was selected as the reference region. The study was carried out in elderly females with different bone mineral densities (normal, osteopenia, and osteoporosis). Quantitative parameters were extracted from the pharmacokinetic model. Parameter *K* ^trans,BM^ (contrast agent extravasation rate constants for blood perfusion of the bone marrow) showed a significant reduction in subjects with lower bone mineral density, which is consistent with previous studies. However, muscle perfusion parameters remained unchanged among different groups. The results indicated that the muscle-based model was stable for bone marrow perfusion modeling. Additionally, nonsignificant change in muscle parameters indicated that the diminished perfusion is only a local rather than a systematic change in the bone marrow for osteoporosis.

## 1. Introduction

Osteoporosis is a common metabolic bone disorder in elderly and its consequence has become one of the most increasing health concerns [1, 2]. When bone mineral content loss and structural deterioration proceed until bone strength becomes sufficiently poor, the cancellous bone increases susceptibility to fracture. As a result, the osteoporotic state has been reached. Studies in recent decades showed a link between peripheral vascular disease and osteoporosis in terms of clinics, epidemiology, and histology [3–5]. Such association indicated a varied blood supply mechanism in the osteoporotic bone. Technically, the microcirculation can be reflected by the dynamic contrast enhanced (DCE) MRI, which provides a direct measurement of tissue perfusion in a living system [6, 7]. Bone marrow perfusion is a physiological process, which can be affected by multiple factors, such as tissue blood flow, capillary capacitance and permeability, interstitial diffusion, interstitial space volume, and venous return [8, 9].

Bone perfusion by DCE-MRI can be assessed by semiquantitative method [10–14] and pharmacokinetic model [15–18]. The former method provides a direct measure of the bone perfusion with semiquantitative parameters derived from the perfusion curves. In comparison, the latter approach has a more complex process, including modeling and curve fitting, but the derived model parameters directly related to inherent physiology. Most pharmacokinetic models include the arterial input function (AIF) [19], such as Tofts model [9]. They adopt the AIF as the input of the tissue perfusion so that the variation in the AIF will change the pharmacokinetics in the tissue. Therefore, the results from such studies depend much on the accuracy of the AIF modeling and the experiment protocol. Brix model [16] is a pharmacokinetic model without using AIF and has been introduced in the bone perfusion study [20]. However, it assumes that the AIF has a fixed pattern, which may not reflect the true physiological process. As a consequence, it is difficult to compare the perfusion function across different studies.

In order to have a more robust analysis, we introduced a new pharmacokinetic model for analyzing bone marrow perfusion, which employed a well-characterized reference region instead of AIF. The erector spinae muscle around the vertebral body was selected as the reference region because its big muscle bulk is easy to be recognized in the images and would be able to derive the DCE-MRI signal with a good signal-to-noise ratio (SNR). The perfusion function of the muscle was quantified by a proposed model to obtain analytic results. The purpose of this study is to characterize bone marrow perfusion properties in subjects with different bone mineral density (BMD) by a muscle-based pharmacokinetic model.

## 2. Methods

### 2.1. Subjects

In order to avoid gender influence, only female subjects were included in current study. The investigation involved a reassessment of DCE-MRI raw data obtained in one previous study [11]. Subjects were excluded if they had (a) clinical or imaging evidence of renal osteodystrophy or other metabolic bone diseases other than osteoporosis or a known malignancy, (b) a history of lumbar spinal surgery or irradiation, or (c) MR imaging evidence of large intravertebral disk herniation, hemangioma, or moderate-to-severe vertebral fracture of L3. Finally, 76 subjects (age 72.5 ± 3.4 years) in total were involved in this retrospective study. The study was approved by the Ethics Committee, Chinese University of Hong Kong, with all participating subjects providing written consent.

### 2.2. Data Acquisition

**Figure 1:**
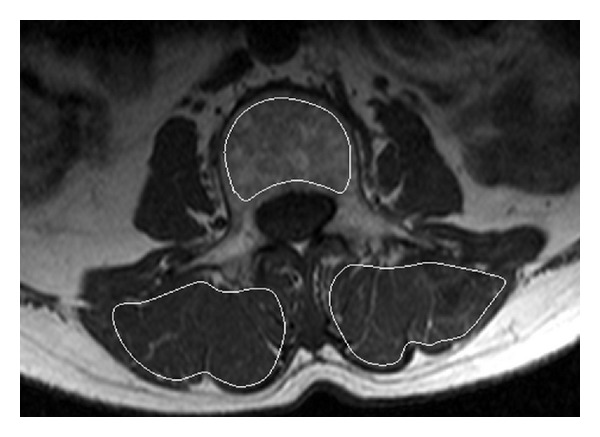
T1-weighted MR image in axial plane (from a subject with normal BMD). The image shows manually drawn ROI positioned within cortical margins of L3 vertebral body and erector spinae muscle for time-signal intensity data points measured from dynamic contrast enhanced images.

Area bone mineral density (BMD) of L3 level was measured by the dual-energy X-ray absorptiometry (DXA). MR imaging was performed at a 1.5 T whole body imaging system (Intera NT; Philips Medical Systems, Best, The Netherlands) with a maximum gradient strength of 30 mT/m. Axial T1-weighted (TR/TE, 450/11 ms; 4 mm thick) MR image of the mid-L3 vertebrae was obtained (shown in Figure 1). Dynamic contrast enhancement MRI (DCE-MRI) data were acquired through the mid-L3 vertebral body region. Dynamic MR imaging was performed using a short T1-weighted gradient-echo sequence (TR/TE, 2.7/0.95 ms; prepulse inversion time, 400 ms; flip angle, 15°; section thickness, 10 mm; number of slice, one; field of view, 250 mm; acquisition matrix, 256 × 256; number of signals acquired, one). A bolus of gadoteric acid (Dotarem, Guerbet, Aulnay, France) at a concentration of 0.15 mmol per kilogram body weight was injected via a power injector (Spectris; Medrad, Indianola, PA, USA) at a rate of 2.5 mL/s through a 20-gauge antecubital vein intravenous catheter (Angiocath; Infusion Therapy Systems, Sandy, UT, USA). Injection was followed by a 20 mL saline flush. Dynamic MR imaging started at the same time the contrast medium injection started. A total of 160 dynamic images were obtained with a temporal resolution of 543 ms, resulting in a total scanning time of 87 seconds.

### 2.3. Data Processing and Modeling of DCE Data

In order to extract the signal intensity curves pixel by pixel, region of interest (ROI) was drawn manually for muscle bulk area of erector spinae and bone marrow area on axial image. The ROIs were drawn following the contour of the erector spinae muscle on both sides and encompassing trabecular bone of vertebral body (as shown in Figure 1). The time-signal intensity curve was generated by averaging signal intensity within each ROI.

**Figure 2:**
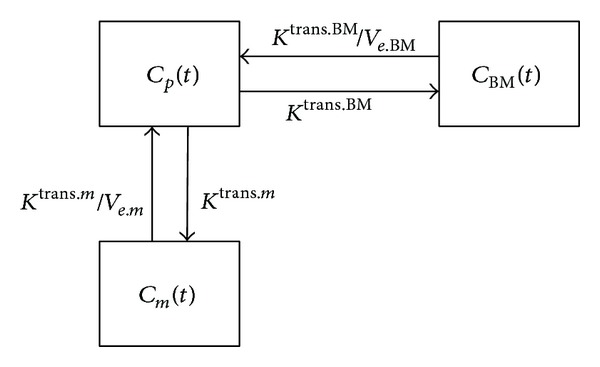
Muscle-based pharmacokinetic modeling scheme. *C*_*p*_, *C*_BM_, and *C*_*m*_ are contrast agent concentrations in the artery, bone marrow, and erector spinae muscle, respectively.

A muscle-based pharmacokinetic model was established, which was modified from a reference region (RR) model [21, 22], to analyze the DCE-MRI data of bone marrow. The model contains three compartments, plasma, muscle, and bone marrow, as shown in Figure 2. When contrast agent is injected into the vessel system, it will go throughout the body with the fluid dynamics, where *C*_*p*_ is the concentration in the supplying artery of the local area for lumbar. By perfusion process, the contrast agent will reach bone marrow and surrounding muscles simultaneously with concentration of *C*_BM_ and *C*_*m*_, respectively. Contrast agent perfusion is modeled by extravasation rate constants, *K* ^trans⁡.BM^ and *K* ^trans⁡.*m*^, as in bone marrow and muscle, respectively. The perfusion space is modeled as extravascular-extracellular volume fraction, which is *v*_*e*.BM_ and *v*_*e*.*m*_ for bone marrow and muscle, respectively. The model describes association of the contrast agent concentration among three compartments, that is, plasma, bone marrow, and muscle.

Based on the modeling structure shown in Figure 2, the contrast agent concentration in bone marrow and muscle can be formulated as the following differential equations:

(1) dCm(t)dt=Ktrans⁡.m·Cp(t)−(Ktrans⁡.mve.m)·Cm(t)

(2) dCBM(t)dt=Ktrans⁡.BM·Cp(t)−(Ktrans⁡.BMve.BM)·CBM(t).

It is obvious that both *C*_*m*_(*t*) and *C*_BM_(*t*) are dependent on *C*_*p*_(*t*), which represents AIF. Merging the two equations will balance out *C*_*p*_(*t*) so that we can formulate the relationship between *C*_BM_(*t*) and *C*_*m*_(*t*) as

(3) CBM(t)=Ktrans⁡.BMKtrans⁡.mCm(t) +(Ktrans⁡.mve.m−Ktrans⁡.BMve.BM)·Cm(t) ⊗exp⁡(−Ktrans⁡.BMve.BM),

which can be simplified as

(4) CBM(t)=R·Cm(t)+R·[(Ktrans⁡.mve.m)−(Ktrans⁡.BMve.BM)]   ·  ∫0tCm(τ)·(exp⁡(−Ktrans⁡.BM)ve.BM)(t−τ)dτ,

where *R* ≡ *K* ^trans⁡.BM^/*K* ^trans⁡.*m*^.

The blood supplying arteries for lumbar vertebra, normally called segmental arteries, are originated from the abdominal aorta. They surround the vertebral body in axial plane and transport the blood to the vertebral marrow and spinal cord by some branches, where some other branches supply the blood to the paravertebral muscles, such as the erector spinae. Because the vertebrae and its surrounding muscles are supplied by the same segmental artery, *C*_*p*_ would influence *C*_BM_ and *C*_*m*_ simultaneously. Based on such association, relationship between the pharmacokinetics of bone marrow and surrounding muscle can be formulated by balancing out the AIF. In other words, it is possible to derive the physiological parameters in bone marrow by taking the muscle as the reference. Therefore, the established muscle-based model (4) can derive the contrast concentration in bone marrow based on that in muscle, which also reflects the contrast dynamics in the artery.

Because DCE-MRI employs fast imaging sequence, the image quality is poor. For extracting characteristic curve, normally people would draw ROI to average the signal intensity of all the included pixels. For AIF acquisition, the area of the artery is too small to get a quality DCE signal. On the other hand, AIF is sensitive to the contrast injection procedure, which also makes the AIF variable. In contrast, the area of muscle bulk is larger to derive a quality DCE signal, which will be easier for analysis. In addition, the pharmacokinetics in muscle is the result after the interaction between the muscle and the artery so that it is not that sensitive to the contrast injection procedure. Therefore, the DCE-MRI signal in muscle is much more stable than in the artery. As a result, the muscle-based modeling would provide a more robust analysis for bone marrow perfusion.

In order to get an analytical solution of (4), an exponential model is employed to formulate the contrast concentration in erector spinae muscle *C*_*m*_(*t*), as shown in the following:

(5) Cm(t)=A·t·exp⁡(−t·B)+C[1−exp⁡(−t·D)]·exp⁡(−t·E),

where *A*, *B*, *C*, *D*, and *E* are density and time constants, respectively. By substituting (5) into (4), the contrast concentration in the bone marrow can be finally formulated as

(6) CBM(t)  =R·Cm(t)+R·[(Ktrans⁡.mve.m)−(Ktrans⁡.BMve.BM)]·f(t),

where the *f*(*t*) is formulated as

(7) f(t)=[A·B2·t2−2A·exp⁡(B·t)−2A·B·t+2A][B3·ve.BM·exp⁡(Ktrans⁡.BM+B·t)] +[C·E·t−C·exp⁡(E·t)+C][E2·ve.BM·exp⁡(Ktrans⁡.BM+E·t)] +[C·D·t−C·exp⁡(t·D+t·E)+C·E·t+C][ve.BM·exp⁡(Ktrans⁡.BM+D·t+E·t)(D+E)2] +[C·t/exp⁡⁡(E·t)−C·t  ][E·ve.BM·exp⁡(Ktrans⁡.BM)] +[C·t/exp⁡(t·D+t·E)−C·t][ve.BM·exp⁡(Ktrans⁡.BM)(D+E)] −[A·B·t2−A·t·exp⁡(B·t)+A·t][B2·ve.BM·exp⁡(Ktrans⁡.BM+B·t)].

**Figure 3:**
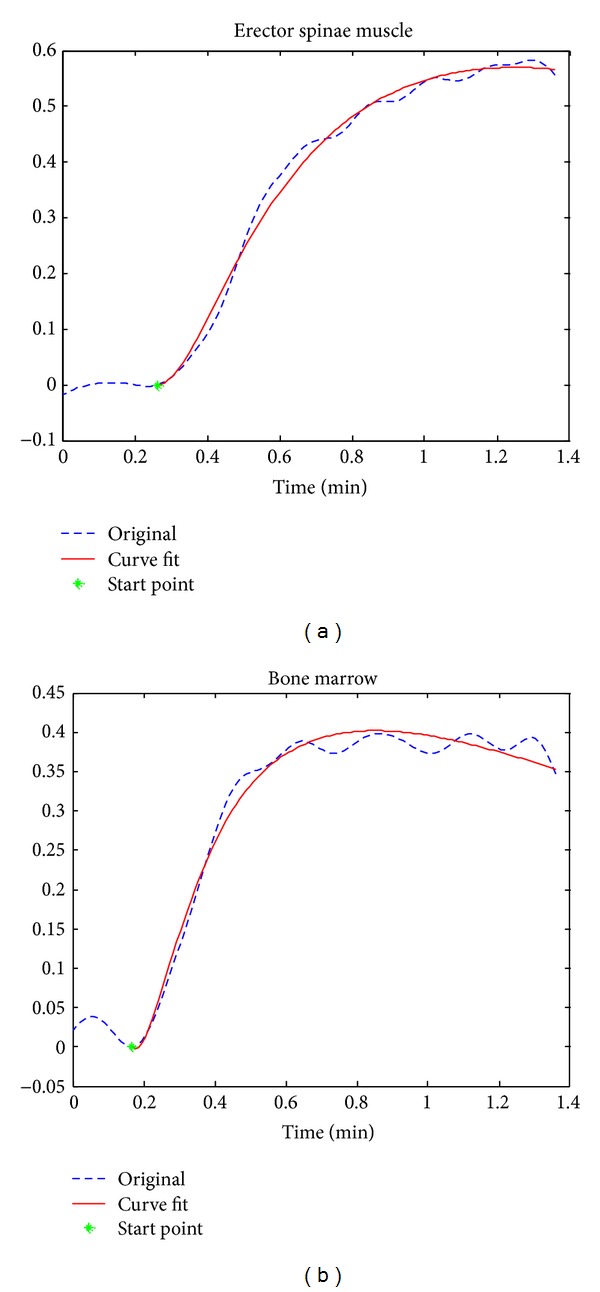
Data processing on DCE data from a subject with normal BMD. (a) Erector spinae muscle curve fitting by muscle-based model to derive characteristic parameters; (b) bone marrow curve fitting by (1).

For each data set, the coefficients in the muscle model (5) were first derived by fitting the DCE-MRI signal from muscle ROI with (5). Then, the fixed coefficients were substituted into (6) to derive the pharmacokinetic parameters, *K* ^trans⁡.BM^, *K* ^trans⁡.*m*^, *v*_*e*.BM_, and *v*_*e*.*m*_, by fitting the DCE-MRI signal of bone marrow. The curve fitting was performed on the DCE-MRI signal with the time course from the starting point to the end of the signal.  Figure 3 shows examples of the curve fitting for the DCE-MRI signals from muscle and bone marrow, respectively. The curve fitting was conducted by using the least square method. In total, 304 parameters of 76 subjects were analyzed. The subjects were classified into three groups according to the T-score derived from the BMD and World Health Organization criteria.

### 2.4. Statistical Analysis 

The investigated subjects were classified into three groups (normal, osteopenia, and osteoporosis) according to their BMD. The statistical descriptions of the model parameters were derived by curve fitting for each subject. Then, the data were compared across the three BMD groups. Analysis of variance method (ANOVA) was employed to evaluate differences in parameters among groups. Statistical analysis was performed using statistical software (SPSS 13.0). A *P* value of less than 0.05 was considered statistically significant.

## 3. Results

Based on the pharmacokinetic model, the perfusion process can be quantitatively analyzed.  Table 1 shows the comparison results by ANOVA analysis. From Table 1, it can be observed that *K* ^trans⁡.BM^ reduces gradually and significantly (*P* = 0.009) in osteopenia and osteoporosis groups compared to the group with normal BMD. For *v*_*e*.BM_, the gradual reduction with the decreasing BMD can be observed but the change does not reach the significant level (*P* = 0.637). However, for the muscle pharmacokinetic parameters, *K* ^trans⁡.*m*^ and *v*_*e*.*m*_ have no significant difference among three groups and no changing trend among the groups.

It indicates that the extravasation rate is significantly reduced during the bone loss process. In other words, the lower the BMD is, the slower the exchange rate in the fluid perfusion from the capillary to the bone marrow is. Although the extravascular-extracellular volume fraction of bone marrow is also decreased in the subjects with lower BMD, it is statistically nonsignificant. While it is interesting to find out that neither extravasation rate nor extravascular-extracellular volume fraction has significant change in erector spinae muscle among the groups with different BMD, further, there is no changing trend of the muscle pharmacokinetic parameters with the reduction of the BMD. It may imply that, with the bone loss process, the surrounding muscle still keeps a normal perfusion function.

## 4. Discussions

With the development of medical imaging, perfusion in tissue can be assessed by DCE-MRI, which, in recent years, has been used to study bone perfusion in a variety of physiological and disease conditions [13, 17, 23–26]. Further, pharmacokinetic models have been employed to analyze bone perfusion function in patients with multiple myeloma, bone edema, and Paget's disease of bone [17, 18, 23]. Bone is composed of trabecular and cortical bone. All of the trabecular bone and the inner two-thirds of the cortical bone receive their blood supply from the marrow cavity [27]. Taking advantage of DCE-MRI, some studies have shown how perfusion parameters are reduced in osteoporotic bone [10–12]. After a bolus injection, tissue concentration of gadolinium is determined by local blood flow, capillary capacitance, vessel permeability, interstitial space, and interstitial diffusion [8]. This is the first study to investigate bone perfusion of osteoporosis by a pharmacokinetic model without directly using AIF.

Most previous work employed Tofts model to assess the bone perfusion [14, 17, 18], of which the analysis of the bone perfusion was much dependent on AIF. It is good to involve the contrast dynamics in the artery when analyzing the perfusion in the tissue. However, because of the small area of vessel in the image and the pulsation of the blood, AIF is quite variable even under the same experiment protocol [21]. Some work used Brix model to assess the bone perfusion [19, 20], where AIF was not included. Such model assumes the MR signal intensity is linearly proportional to the concentration of contrast agent. This assumption may not hold true for different experiment conditions. Therefore, establishing a pharmacokinetic modeling scheme, which is reliable and stable enough but still reflects the contrast dynamics in the vessel, will provide a more precise assessment of perfusion function. The proposed muscle-based model achieved such purpose for investigating bone perfusion.

Firstly, the parameter *v*_*e*.BM_ was observed to be reduced with a decreased BMD, which indicated a reduced capacity for blood perfusion in osteoporotic bone. Increased marrow fat in osteoporotic bone may be one reason for the decreased extravascular-extracellular volume fraction in bone marrow. A previous work reported an increased marrow fat content in the osteoporosis patients by using MR spectroscopy [28]. Our previous studies for osteoporosis also supported this finding [10, 20]. In osteoporotic bone, the increased marrow fat would reduce interstitial space for perfusion, which could result in a diminished extravascular-extracellular volume fraction [10–12, 28, 29]. However, the change in *v*_*e*.BM_ among the three groups was nonsignificant, indicating that the marrow fat content change may not be the main contribution to the perfusion function degeneration in osteoporosis.

Secondly, *K* ^trans⁡.BM^ was also found decreased in subjects with lower BMD, implying a degenerated blood supply function. Such degeneration could also diminish the nutrition exchange between the bone tissue and the artery. It appears that the exchange rate across the vessel wall is reduced as BMD decreases. Multiple factors can affect this exchange rate, such as capillary endothelial permeability and interstitial or intraosseous pressure. For the latter factor, higher interstitial pressures will limit diffusion of molecules between the capillary bed and the interstitial space. It has been revealed by a previous study [30] that increased marrow fat increases intraosseous pressure. The increased marrow fat content would limit exchange between the intravascular and interstitial spaces resulting in a weakened perfusion rate. Reduction of arterial capillary density could be another reason for the decreased *K* ^trans⁡.BM^ in the bone with lower BMD. Patients with proximal femoral osteoporosis have been reported with reduced density of arterial capillaries and more frequent arteriosclerotic vascular lesions [31]. Another study on multiple myeloma infiltration of vertebral bodies found that the reduction of the blood volume during the perfusion mirrored bone marrow vessel density assessed histologically [23].

Thirdly, it is interesting that muscle perfusion indices did not change with BMD (*P* > 0.6). With respect to vascular inflow, previous studies have shown that the perfusion anomalies occurring in osteoporosis most likely originate within bone but not within adjacent muscle [11, 12]. In other words, decreased perfusion function in osteoporosis is a local degeneration of bone rather than a systematic circulatory disturbance.

This retrospective study had one main limitation. The data acquisition duration was relatively short at 87 seconds, which may limit the assessment of the full wash-out phase and thus the influence the parameter derivation. The derived parameters may be deviated from the true value. However, the trend for the parameter change among different groups still reflects the real situation and is supported by other studies.

In conclusion, a muscle-based pharmacokinetic model was proposed for bone marrow perfusion without using AIF. Such model avoided the direct association with the variable contrast agent dynamics in the artery and could provide a more reliable analysis. The perfusion indices, *v*_*e*.BM_ and *K* ^trans⁡.BM^, were both decreased in osteoporotic bone. Decreased interstitial space and reduced capillary density are possible reasons for the degenerated perfusion function. Further, these factors should be considered in the mechanism investigation of osteoporosis.

## Figures and Tables

**Table 1:** Comparison among groups.

Parameter	Group (*N*)	Mean	SD	*P* value
*K* ^trans.BM^ (min^−1^)	Normal (*n* = 11)	0.369	0.129	0.009
	Osteopenia (*n* = 26)	0.334	0.110	
	Osteoporosis (*n* = 39)	0.255	0.135	

*K* ^trans.*m*^ (min^−1^)	Normal (*n* = 11)	0.303	0.101	0.623
	Osteopenia (*n* = 26)	0.310	0.116	
	Osteoporosis (*n* = 39)	0.280	0.136	

*v*_*e*.BM_	Normal (*n* = 11)	0.191	0.245	0.637
	Osteopenia (*n* = 26)	0.146	0.214	
	Osteoporosis (*n* = 39)	0.125	0.184	

*v*_*e*.*m*_	Normal (*n* = 11)	0.186	0.286	0.662
	Osteopenia (*n* = 26)	0.114	0.177	
	Osteoporosis (*n* = 39)	0.159	0.276	
